# SUN2 Modulates HIV-1 Infection and Latency through Association with Lamin A/C To Maintain the Repressive Chromatin

**DOI:** 10.1128/mBio.02408-17

**Published:** 2018-05-01

**Authors:** Wei-Wei Sun, Shi Jiao, Li Sun, Zhaocai Zhou, Xia Jin, Jian-Hua Wang

**Affiliations:** a CAS Key Laboratory of Molecular Virology and Immunology, Institut Pasteur of Shanghai, Chinese Academy of Sciences, Shanghai, China; b University of Chinese Academy of Sciences, Beijing, China; c State Key Laboratory of Cell Biology, Institute of Biochemistry and Cell Biology, Shanghai Institutes for Biological Sciences, Chinese Academy of Sciences, Shanghai, China; University of North Carolina at Chapel Hill; Columbia University

**Keywords:** HIV-1 latency, Sad1 and UNC84 domain containing 2, long-terminal repeat, repressive chromatin

## Abstract

The postintegrational latency of HIV-1 is characterized by reversible silencing of long terminal repeat (LTR)-driven transcription of the HIV genome. It is known that the formation of repressive chromatin at the 5′-LTR of HIV-1 proviral DNA impedes viral transcription by blocking the recruitment of positive transcription factors. How the repressive chromatin is formed and modulated during HIV-1 infection remains elusive. Elucidation of which chromatin reassembly factor mediates the reorganization of chromatin is likely to facilitate the understanding of the host’s modulation of HIV-1 transcription and latency. Here we revealed that “Sad1 and UNC84 domain containing 2” (SUN2), an inner nuclear membrane protein, maintained the repressive chromatin and inhibited HIV LTR-driven transcription of proviral DNA through an association with lamin A/C. Specifically, lamin A/C tethered SUN2 to the nucleosomes 1 and 2 of the HIV-1 5′-LTR to block the initiation and elongation of HIV-1 transcription. SUN2 knockdown converted chromatin to an active form and thus enhanced the phosphorylation of RNA polymerase II and its recruitment to the 5′-LTR HIV-1 proviral DNA, leading to reactivation of HIV-1 from latency. Conversely, the exogenous factors such as tumor necrosis factor alpha (TNF-α) induced reactivation, and the replication of HIV-1 led to the disassociation between SUN2 and lamin A/C, suggesting that disruption of the association between SUN2 and lamin A/C to convert the repressive chromatin to the active form might be a prerequisite for the initiation of HIV-1 transcription and replication. Together, our findings indicate that SUN2 is a novel chromatin reassembly factor that helps to maintain chromatin in a repressive state and consequently inhibits HIV-1 transcription.

## IMPORTANCE

Despite the successful use of scores of antiretroviral drugs, HIV latency poses a major impediment to virus eradication. Elucidation of the mechanism of latency facilitates the discovery of new therapeutic strategies. It has been known that the formation of repressive chromatin at the 5′-LTR of HIV-1 proviral DNA impedes viral transcription and maintains viral latency, but how the repressive chromatin is formed and modulated during HIV-1 infection remains elusive. In this study, we performed in-depth virological and cell biological studies and discovered that an inner nuclear membrane protein, SUN2, is a novel chromatin reassembly factor that maintains repressive chromatin and thus modulates HIV-1 transcription and latency: therefore, targeting SUN2 may lead to new strategies for HIV cure.

## INTRODUCTION

Despite the high effectiveness of combination antiretroviral therapy, the persistence of the HIV-1 viral reservoir represents one of the major obstacles for functional cure (1–4). The reversible silencing of transcription driven by the viral 5′-long terminal repeat (LTR) promoter is the major determinant for the establishment and maintenance of HIV-1 postintegrational latency (3, 5). The chromatin organization of the 5′-LTR of HIV-1 proviral DNA tightly regulates viral transcription. The repressive proviral 5′-LTR is organized into 3 strictly positioned nucleosomes (NUC0, NUC1, and NUC2) separated by two intervening enhancer regions: DNase hypersensitive site 1 (DHS-1) and DHS-2. NUC1, the nucleosome positioned immediately downstream of the LTR transcription start site, is particular repressive to transcription, and its disruption is required for LTR activation (6).

The repressive chromatin or nucleosome can be modulated by different chromatin reassembly factors. The ATP-dependent chromatin remodeling complex SWI/SNF (switch/sucrose nonfermentable)/BAF (SWI/SNF-A) positions the repressive NUC1 for transcription interference (7), and the other chromatin reassembly factors Spt6 (chromatin-specific transcription elongation factor SPT6) and Chd1 (chromodomain-helicase-DNA-binding protein 1) have also been shown to maintain a repressive chromatin state at the proviral 5′-LTR and repress transcription (8, 9). Additionally, the deacetylation modification of histone, the increased trimethylation at histone-3–lysine 27 and lysine 9, along with the CpG methylation, of the proviral 5′-LTR, contribute to the highly restrictive chromatin form (10–18). The specific regulation of chromatin organization, by either regulating the expression of chromatin reassembly factors and their associations with repressive nucleosome or altering the histone epigenetic modifications, modulates HIV-1 proviral 5′-LTR activity and viral latency. However, how the repressive nucleosome or chromatin is formed and modulated during HIV-1 infection remains elusive. To uncover the chromatin reassembly factor that mediates reorganization of chromatin is likely to facilitate the understanding of how the host modulates HIV-1 transcription and latency.

SUN2 is an inner nuclear membrane protein, accompanying other nuclear envelope proteins such as SUN1, nesprin-1, and nesprin-2 to form the linker between nucleoskeleton and cytoskeleton (LINC) complexes (19–21). The LINC complexes provide the structural support to the nucleus and physically couple the nucleoskeleton with the cytoskeleton. They are involved in the mechanotransduction function and regulations of cellular signaling and gene expression. Mutations in genes encoding LINC complex proteins are with cardiac and skeletal myopathies (22, 23).

The LINC components interact with each other and other binding partners at the nuclear envelope to perform their functions. The carboxyl terminus of SUN2 is detained in the perinuclear space to bind other nuclear envelope proteins, such as nesprin-1 and nesprin-2, through linking with their Klarsicht, Anc-1, Syne-1 homology (KASH) domains (20, 21). The amino terminus of SUN2 extends into the nucleoplasm and associates with lamins (24), enabling its anchoring to the nuclear envelope (25). Lamins provide the structural integrity for cytoskeletal organization and nuclear positioning, as well as anchoring sites for chromatin domains. By association with numerous host proteins located at the nuclear periphery and in the nucleoplasm, lamins regulate genome organization, gene expression, and cellular signaling (26–32).

The overexpression of SUN2 has previously been reported to inhibit HIV-1 infection in cell lines and primary monocyte-derived dendritic cells (MDDCs) (33, 34). Whether the endogenous SUN2 also modulates HIV infection remains controversial (33, 35). In this study, we provide novel insights into the mechanisms by which the endogenous SUN2 mediates the inhibition of HIV-1 infection. SUN2 repressed HIV-1 LTR promoter-driven gene expression through an association with lamin A/C and the maintenance of repressive chromatin. Specifically, lamin A/C tethers SUN2 to NUC1 and NUC2 positions and inhibits both the initiation and elongation of HIV-1 transcription. These results demonstrate that SUN2 is a novel chromatin reassembly factor that modulates HIV-1 proviral DNA transcription.

## RESULTS

### SUN2 inhibits HIV-1 infection by suppressing viral transcription.

**FIG 1:**
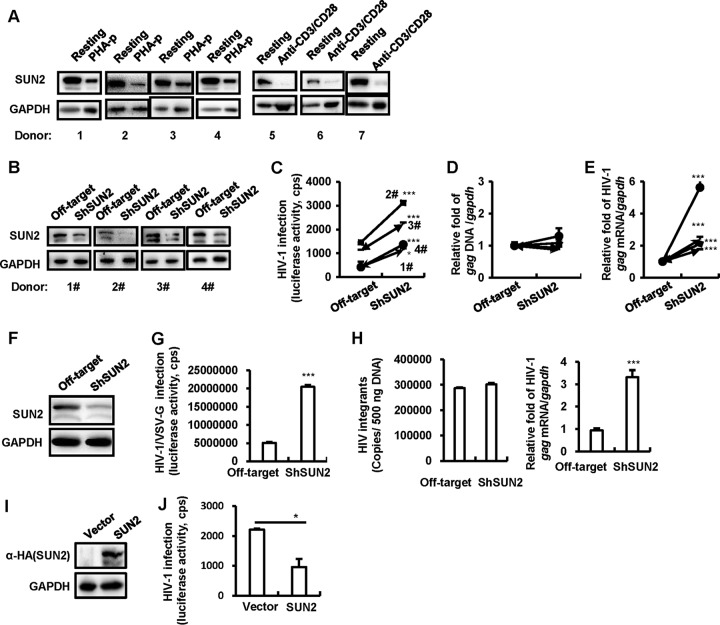
SUN2 inhibits HIV-1 infection by suppressing viral transcription. (A) Resting CD4^+^ T cells (1 × 10^6^) were activated with or without 5 µg/ml of PHA-P or anti-CD3/CD28 antibody-coated microbeads for 3 days, and then the cells were harvested, and endogenous expression of SUN2 in primary CD4^+^ T cells was detected by Western blotting. (B to E) SUN2 knockdown increased HIV-1 replication and transcription in primary CD4^+^ T cells. PHA-P-activated primary CD4^+^ T cells (1 × 10^6^) were infected with lentivirus vectors containing SUN2-specific shRNA or the off-target control for 72 h, and then SUN2 expression was detected by Western blotting (B); the same cells were further infected with single-cycle infectious HIV-luc/NL4-3 (5 ng p24^*gag*^) for an additional 72 h, and viral replication was quantified by detecting luciferase activity (C). Cellular DNA and mRNA were isolated, and the integrated proviral DNA and transcriptional mRNA were quantified by detection of *gag* DNA and mRNA levels, respectively (D and E). (F to H) SUN2 knockdown increased HIV-1 replication and transcription in Jurkat T cells. (F) SUN2 expression in Jurkat T cells (1 × 10^6^) was knocked down and detected by immunoblotting as described above. (G and H) Cells were further infected with HIV-luc/VSV-G (5 ng p24^*gag*^) for an additional 48 h, viral infection was detected by measuring luciferase activity (G), and the integrated proviral DNA and transcriptional mRNA were quantified. The integrated HIV-1 proviral DNA was quantified using a two-step Alu PCR (H). (I and J) SUN2 overexpression inhibited HIV-1 infection. Jurkat T cells were transfected with pCDNA3.1-HA/SUN2 (or vector control) plasmid for 48 h and then infected with HIV-luc/NL4-3 (5 ng p24^*gag*^) for an additional 48 h. The SUN2 expression was detected by Western blotting (I). The viral infection was detected by measuring luciferase activity (J). Data are presented as mean ± standard deviation (SD). *P* < 0.05 (*) and *P* < 0.001 (***) were considered significant differences in a Wilcoxon signed-rank test. cps, counts per second.

We first investigated the endogenous expression of SUN2 in primary CD4^+^ T cells. SUN2 showed high expression in the resting CD4^+^ T cells isolated from healthy donors but diminished expression upon stimulations with phytohemagglutinin-P (PHA-P) or anti-CD3/CD28 antibodies (Fig. 1A).

SUN2 has previously been reported as the product of an interferon-stimulated gene (ISG) with anti-HIV-1 activity (34). To confirm its inhibitory role on HIV-1 infection, SUN2 expression was knocked down by infection with lentiviruses containing shSUN2 (i.e., SUN2 short hairpin RNA [shRNA]) for 3 days in PHA-P-activated primary CD4^+^ T cells (Fig. 1B). These cells were then infected with a single-cycle infectious HIV-luc/NL4-3 virus for an additional 3 days. Results showed that SUN2 knockdown significantly increased HIV-1 infection (Fig. 1C).

Further analyses revealed that SUN2 affected HIV-1 postintegrational steps, as the integrated proviral DNA quantified with *gag* PCR showed similar levels in SUN2 knockdown cells and cells transfected with an off-target control (Fig. 1D); when quantifying the production of HIV-1 *gag* mRNA in these primary CD4^+^ T cells, SUN2 knockdown significantly increased the expression of *gag* mRNA, suggesting that SUN2 repressed the transcription of HIV-1 proviral DNA (Fig. 1E).

The 5-day transduction of shSUN2 to silence the endogenous SUN2 in activated primary CD4^+^ T cells may affect HIV infection indirectly through impairment of cellular function (36). To rule out this possibility, we performed a 3-day transduction of shSUN2 and then infected cells with HIV-1 for an additional 3 days and found that the shRNA transduction did not change the T-cell activation status after the total 6-day incubation, by monitoring the surface expression of CD25 and HLA-DR (see Fig. S1 in the supplemental material).

10.1128/mBio.02408-17.1 FIG S1  Cell activation assay. PHA-P- or anti-CD3/CD8 antibody cocktail-treated primary CD4^+^ T cells (1 × 10^6^) were transduced with or without lentiviruses containing SUN2 shRNA or the off-target control for 72 h, and then cells were further infected with HIV-luc/NL4-3 (5 ng p24^*gag*^) for an additional 72 h. Cells were harvested for immunostaining with specific antibodies and detected by flow cytometry. The percentage of positive staining is indicated. Download FIG S1, TIF file, 1 MB. Copyright © 2018 Sun et al. 2018 Sun et al. This content is distributed under the terms of the Creative Commons Attribution 4.0 International license.

To prepare for detailed mechanistic studies, we performed a similar assessment of the inhibitory role of SUN2 on HIV-1 infection in the Jurkat CD4^+^ T-cell line. SUN2 knockdown significantly increased the infection of HIV-luc/VSV-G (Fig. 1F and G) and elevated the production of HIV-1 *gag* mRNA but kept HIV-1 integration at a similar level to that in the off-target controls (Fig. 1H). Although the double knockout of *Sun2* and *Sun1* in mouse embryonic fibroblasts has been shown to induce premature proliferation and increase apoptosis (37), in our system, the knockdown of SUN2 alone in Jurkat T cells did not markedly affect cell viability, as over 74% of cells remained viable (see Fig. S2 in the supplemental material). The human *Sun2* gene encoding the full length of the 717-amino-acid protein was cloned into the pcDNA3.1 plasmid with a C-terminal hemagglutinin (HA) tag. SUN2 overexpression significantly inhibited the infection of HIV-luc/NL4-3 virus in Jurkat T cells (Fig. 1I and J). Taken together, these data demonstrate that SUN2 inhibits HIV-1 infection by suppressing the transcription of proviral DNA.

10.1128/mBio.02408-17.2 FIG S2  Cell viability assay. Jurkat T cells (1 × 10^6^) were infected with the lentiviruses containing SUN2-specific shRNA or the off-target control for 72 h, and cell viability was monitored by staining with anti-annexin-V–FITC antibody and propidium iodide (PI) and then analyzed by flow cytometry. Download FIG S2, TIF file, 0.1 MB. Copyright © 2018 Sun et al. 2018 Sun et al. This content is distributed under the terms of the Creative Commons Attribution 4.0 International license.

### SUN2 suppresses HIV-1 LTR-driven gene expression.

**FIG 2:**
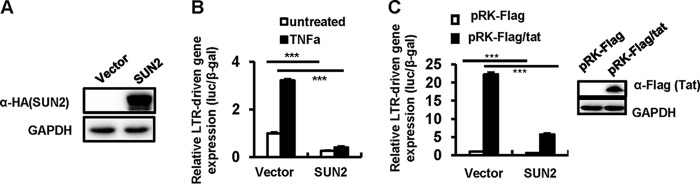
SUN2 suppresses HIV-1 LTR-driven gene expression. HEK293T cells were cotransfected with pCDNA3.1-HA/SUN2 (or vector control) plasmid, which contains an HIV-1_NL4-3_-LTR promoter-driven luciferase reporter, with or without pRK-Flag/tat, for 24 h; the β-galactosidase (β-Gal)-expressing vector pCMV-β-galactosidase was used to normalize transfection efficiency, and then cells were treated with or without TNF-α (5 ng/ml) for an additional 24 h. SUN2 overexpression was detected by Western blotting (A), and reporter gene expression was assessed by luciferase assay (B and C). Data are presented as mean ± SD. Results are representative of four independent experiments. ***, *P* < 0.001 as determined by an unpaired *t* test.

The HIV-1 LTR promoter plays an essential role in driving viral transcription and productive infection (38, 39). To further determine the mechanism of SUN2-mediated inhibition of HIV-1 transcription, we investigated whether SUN2 could inhibit LTR activity by cotransfection of HEK293T cells with the SUN2-expressing pcDNA3.1 plasmid along with a luciferase reporter driven by the full-length LTR promoter from HIV-1_NL4-3_ and then treated the transfected cells with or without tumor necrosis factor alpha (TNF-α), which is known to enhance LTR activity (40). We observed that the overexpression of SUN2 (Fig. 2A) significantly inhibited LTR-driven basal gene expression by 2.0-fold (*P* < 0.001) and TNF-α stimulated gene expression by 3.3-fold (*P* < 0.001) (Fig. 2B).

Because HIV-1 Tat protein binds to the transactivation response (TAR) element to drive transcription elongation, we next investigated whether SUN2 could suppress Tat-driven transactivation of transcription by cotransfection of HEK293T cells with an HIV-1 *tat*-expressing plasmid (pRK-Flag/tat, cloned from the HIV-1_NL4-3_ B subtype) and pCDNA3.1/SUN2, as well as a plasmid containing the LTR. We observed that the overexpression of SUN2 significantly inhibited Tat-driven LTR activity by about 4-fold (*P* < 0.001) (Fig. 2C). Taken together, these data demonstrate that SUN2 suppresses HIV-1 LTR-driven gene expression.

### SUN2 knockdown increases HIV-1 reactivation from proviral DNA.

**FIG 3:**
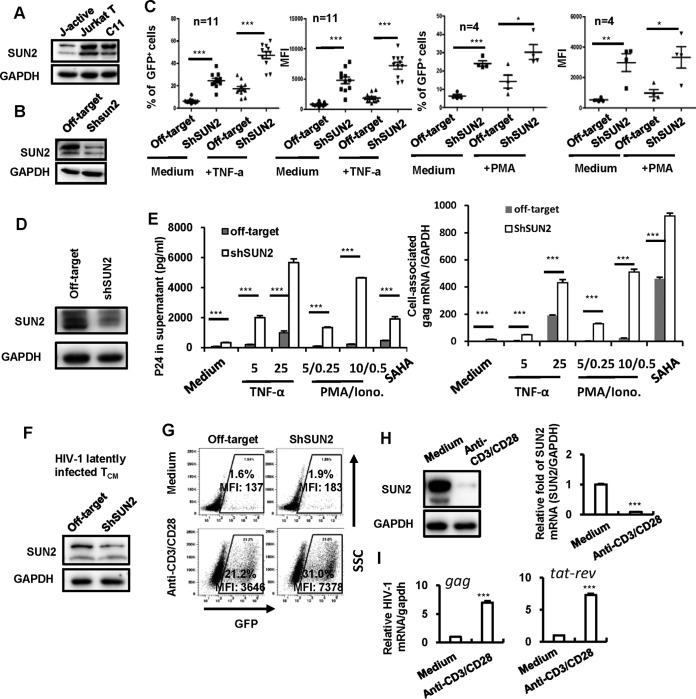
SUN2 knockdown increases HIV-1 reactivation from an integrated HIV-1 proviral DNA. (A to C) SUN2 knockdown promoted HIV-1 reactivation in C11 cells. (A) SUN2 expression in uninfected or HIV-1 latently (C11) or actively (J-active) infected Jurkat T cells detected by Western blotting. (B) C11 cells were infected with lentiviruses containing SUN2 shRNA or the off-target control for 72 h, and SUN2 knockdown was determined by Western blotting. (C) Cells were stimulated with or without TNF-α (1 ng/ml) or PMA (1 nM) for an additional 24 h, HIV-1 reactivation was measured by detecting GFP expression, and the percentage of GFP^+^ cells and mean fluorescence intensity (MFI) were calculated. Results from several repeats are summarized. (D and E) SUN2 knockdown promoted HIV-1 reactivation in latently infected ACH2 cells. ACH2 cells were infected with lentivirus vectors containing SUN2 shRNA or the off-target control for 72 h, SUN2 knockdown was determined by Western blotting (D), and cells were stimulated with or without TNF-α or PMA-ionomycin for an additional 24 h, viral reactivation was detected by quantifying the produced viral particles in supernatant with p24^*gag*^ capture ELISA or the cell-associated *gag* mRNA (E). (F and G) SUN2 knockdown promoted HIV-1 reactivation in latently infected T_CM_ cells. HIV-1 latently infected T_CM_ cells (1 × 10^6^) were infected with lentivirus vectors containing SUN2 shRNA or the off-target control for 72 h. SUN2 knockdown was determined by Western blotting (F). The cells were reactivated with anti-CD3/CD28 antibodies for an additional 3 days, and HIV-1 reactivation was measured by detecting the positive percentage of GFP^+^ cells and their MFI (G). (H and I) The treatment with anti-CD3/CD28 antibodies diminished the endogenous expression of SUN2 in HIV-1 latently infected T_CM_ cells and reactivated HIV-1 from latency. The endogenous expression of SUN2 in HIV-1 latently infected T_CM_ cells stimulated with or without anti-CD3/CD28 antibodies was detected by qPCR and Western blotting (H), and the levels of HIV-1 *gag* and *tat-rev* mRNA were quantified by qPCR (I). *, *P* < 0.05, **, *P* < 0.01, and ***, *P* < 0.001, significantly different as determined by an unpaired *t* test.

The reversible silencing of LTR-driven transcription is critical for an integrated provirus to maintain viral latency (3, 5, 41). The inhibitory effect of SUN2 on HIV-1 LTR-driven gene expression suggests a potential role for SUN2 in maintaining HIV-1 latency. Thus, we sought to determine the effect of endogenous SUN2 on the expression of an integrated HIV-1 proviral DNA in CD4^+^ T cells. The HIV-1 latently infected Jurkat T-cell clone (C11) harboring an HIV-1 proviral DNA encoding green fluorescent protein (GFP) was used (42). These cells can be reactivated upon stimulation with TNF-α, vorinostat (suberanilohydroxamic acid [SAHA]), or trichostatin A to express GFP as an indication of HIV-1 reactivation from latency (42, 43). We observed higher expression of SUN2 in the HIV-1 latently infected Jurkat T-cell clone C11 than in HIV-1 actively infected counterpart cells (J-active cells) (Fig. 3A). The endogenous SUN2 in C11 cells was successfully knocked down by using specific shRNA (Fig. 3B), and cells were measured for HIV-1 reactivation by detection of GFP expression upon stimulation with or without different latency reversal agents (LRAs). In the presence or absence of stimulation with TNF-α or phorbol-12-myristate-13-acetate (PMA) (Fig. 3C), SUN2 knockdown significantly increased HIV-1 reactivation. These results suggest that endogenous SUN2-mediated suppression of HIV-1 gene expression from proviral DNA contributes to viral latency in CD4^+^ T cells.

To confirm the observed function of SUN2 on HIV gene expression is not an artifact in a single cell line, we performed the same experiment in ACH2 cell clone which is derived from HIV-1 latently infected CD4^+^ CEM cells, contains a single copy of proviral DNA per cell, and can be induced to produce infectious HIV-1 particles (44, 45). The endogenous SUN2 in ACH2 cells was successfully knocked down by using specific shRNA (Fig. 3D), and then cells were further stimulated with or without TNF-α, PMA-ionomycin, or vorinostat (SAHA). Viral reactivation was detected by quantifying the produced viral particles in supernatant with p24^*gag*^ capture enzyme-linked immunosorbent assay (ELISA) or the cell-associated *gag* mRNA. In the presence or absence of stimulation, SUN2 knockdown significantly increased HIV-1 reactivation (Fig. 3E).

Previous analyses using primary cells from HIV-infected individuals revealed that resting CD4^+^ memory T cells are the main cellular reservoir (46–48) and that central memory T cells (T_CM_ cells) are one of the most important reservoirs (49, 50). To investigate the role of SUN2 in regulating HIV-1 latency in CD4^+^ T_CM_ cells, we used an established method to generate a large number of HIV-1 latently infected human primary CD4^+^ T cells (51), which had been used as an important tool for investigating the mechanisms for the maintenance and reactivation of HIV-1 latency and the signaling pathways involved (52). The endogenous SUN2 in HIV-1 latently infected T_CM_ cells was knocked down by using specific shRNA (Fig. 3F), and the latently infected GFP reporter HIV-1 in these cells was activated with anti-CD3/CD28 antibodies. Compared to the activated control cells, SUN2 knockdown increased HIV-1 reactivation from 21.2% to 31.0%, and there was a 2-fold increase in the mean fluorescence intensity (MFI) in GFP^+^ cells (Fig. 3G). We also observed that the stimulation with anti-CD3/CD28 antibodies dramatically reduced SUN2 expression in HIV-1 latently infected T_CM_ cells and reactivated HIV-1 from latency (Fig. 3H). HIV-1 reactivation was verified by quantifying the production of HIV-1 *gag* and *tat-rev* mRNAs (Fig. 3I). Taken together, these results suggest that SUN2 negatively regulates HIV-1 gene transcription to maintain viral latency in both HIV-1 latently infected cell lines and primary central memory CD4^+^ T cells.

### Lamin A/C tethers SUN2 to the HIV-1 LTR NUC1 and NUC2 regions to inhibit LTR activity.

SUN2 is an inner nuclear membrane protein that plays a major role in nuclear-cytoplasmic connection by formation of a “bridge” across the nuclear envelope (19). The C-terminal domain of SUN2 tethers the KASH (Klarsicht, ANC-1, and Syne homology) domain on the outer nuclear membrane, and the N-terminal domain binds to lamin A/C in the endonuclear space (24).

**FIG 4:**
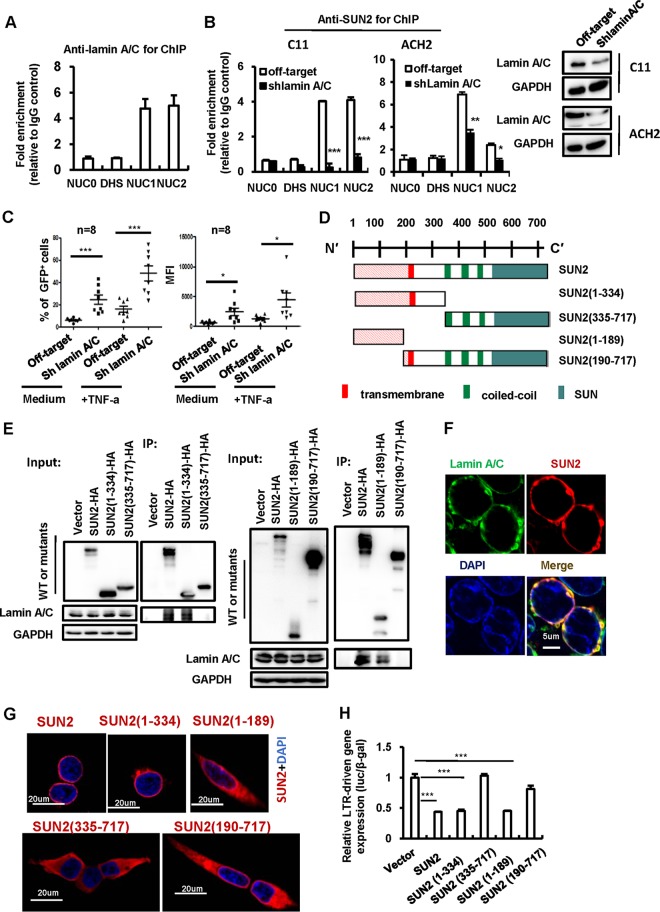
Lamin A/C tethers SUN2 to the HIV-1 LTR NUC1 and NUC2 regions to inhibit LTR activity. (A) The association of endogenous lamin A/C with the HIV-1 5′-LTR regions was determined in C11 cells by a cross-linked ChIP assay. The IgG antibody control was used for normalization. (B) Lamin A/C tethers SUN2 to the HIV-1 5′-LTR. C11 or ACH2 cells were infected with lentiviruses containing lamin A/C shRNA or the off-target control for 72 h, and then the association of endogenous SUN2 with different HIV-1 5′-LTR regions was determined by a cross-linked ChIP assay. The lamin A/C knockdown was assessed by Western blotting (right panel). (C) Lamin A/C knockdown increases HIV-1 reactivation from an integrated HIV-1 proviral DNA. C11 cells with or without lamin A/C knockdown performed as described above were treated with or without TNF-α (1 ng/ml) for an additional 24 h. HIV-1 reactivation was measured by detecting the GFP^+^ cells. The positive percentage of GFP^+^ cells and their MFI were calculated and analyzed. (D) Schematics of the wild-type SUN2 protein motif and its mutants. (E) The N-terminal endonuclear domain of SUN2 mediated its association with lamin A/C. HEK293T cells were transfected with pCDNA3.1-HA/SUN2 or SUN2 mutant plasmids, the whole-cell lysates were prepared, immunoprecipitations were performed with anti-HA antibody, and lamin A/C and GAPDH were immunoblotted with specific antibodies. Results are representative of three independent experiments. (F, G) Cellular location of SUN2 and its mutants. HEK293T cells were seeded onto poly-l-lysine-coated microscope slides and transfected with the indicated plasmids. Anti-HA antibody was used for immunostaining to indicate SUN2 or mutants (red), lamin A/C was imunostained with specific antibodies and indicated in green, and the nucleus was indicated with DAPI staining (blue). Cells were observed under confocal microscopy. (H) Inhibition of HIV-1 LTR activity mediated by SUN2 or its mutants. HEK293T cells were transfected with pCDNA3.1-HA/SUN2 or SUN2 mutant plasmids along with HIV-1_NL4-3_-LTR promoter-driven luciferase reporter plasmid for 48 h, and then the luciferase activity was measured and normalized by β-galactosidase activity. Data are presented as mean ± SD. Results are representative of three independent experiments. *, *P* < 0.05, **, *P* < 0.01, and ***, *P* < 0.001, significantly different as determined by an unpaired *t* test.

Lamin proteins can interact with chromatin and are thought to be involved in modulating nuclear stability and gene expression (53–55). To investigate whether the association of SUN2 with lamin A/C is required for SUN2-mediated inhibition on HIV-1 LTR activity, we first determine the potential binding of lamin A/C with HIV-1 LTR promoter. We performed the chromatin immunoprecipitation (ChIP) analysis in HIV-1 latently infected C11 cells with anti-lamin A/C antibodies and analyzed the products using specific primers targeting HIV LTR NUC0, DHS, NUC1, or NUC2 regions and found the association of lamin A/C with HIV LTR NUC1 and NUC2 regions (Fig. 4A). Simultaneously, we found that SUN2 mainly bound to LTR NUC1 and NUC2 regions in both C11 and ACH2 cells (Fig. 4B) and that the knockdown of lamin A/C significantly impaired SUN2 positioning to these two regions (Fig. 4B), suggesting that lamin A/C served as a bridge for SUN2 binding to HIV-1 LTR. Additionally, the knockdown of lamin A/C promoted HIV-1 reactivation from latently infected C11 cells (Fig. 4C).

To further confirm that the association between SUN2 and lamin A/C is required for SUN2-mediated inhibition of HIV-1 LTR activity, we constructed the C- or N-terminal-truncated mutants of SUN2 plasmids (Fig. 4D) and then transfected them into HEK293T cells individually. Immunoprecipitations with endogenous lamin A/C were performed. The C-terminus deletion [e.g., SUN2(1–334)- and SUN2(1–189)-containing mutants] did not affect the association with the endogenous lamin A/C, while the N-terminus deletion [e.g., SUN2(335–717)- and SUN2(190–717)-containing mutants] lost the association with lamin A/C (Fig. 4E). The association of SUN2 and lamin A/C was also observed under confocal microscope (Fig. 4F).

The deletion of the C-terminal amino acids from 335 to 717 [SUN2(1–334)-containing mutant] did not affect the mutant’s location at the nuclear membrane, as observed under a confocal microscope (Fig. 4G), whereas further deletion of the transmembrane domain-contained region [SUN2(1–189)-containing mutant] changed its distribution in cytoplasm. The N-terminus deletion mutant SUN2(335–717) lost its nuclear membrane location, whereas other SUN2(190–717) mutant still maintained location at the nuclear membrane, due possibly to the existence of a transmembrane domain (Fig. 4G). When we cotransfected these mutant plasmids into HEK293T cells along with the LTR-promoter-containing luciferase reporter, the N-terminus deletion mutants SUN2(335–717) and SUN2(190–717), which lost their association with lamin A/C, despite retaining their nuclear membrane location [SUN2(190–717) mutant], abolished the inhibition of LTR-driven basal gene expression (Fig. 4H).

Taken together, these data demonstrate that lamin A/C tethers SUN2 to HIV-1 LTR regions and the association of SUN2 through its N-terminal endonuclear domain with lamin A/C for location at the nuclear membrane is required for SUN2-mediated inhibition of HIV-1 LTR activity.

### SUN2 maintains the repressive chromatin of HIV-1 5′-LTR and inhibits both the initiation and elongation of HIV-1 transcription.

**FIG 5:**
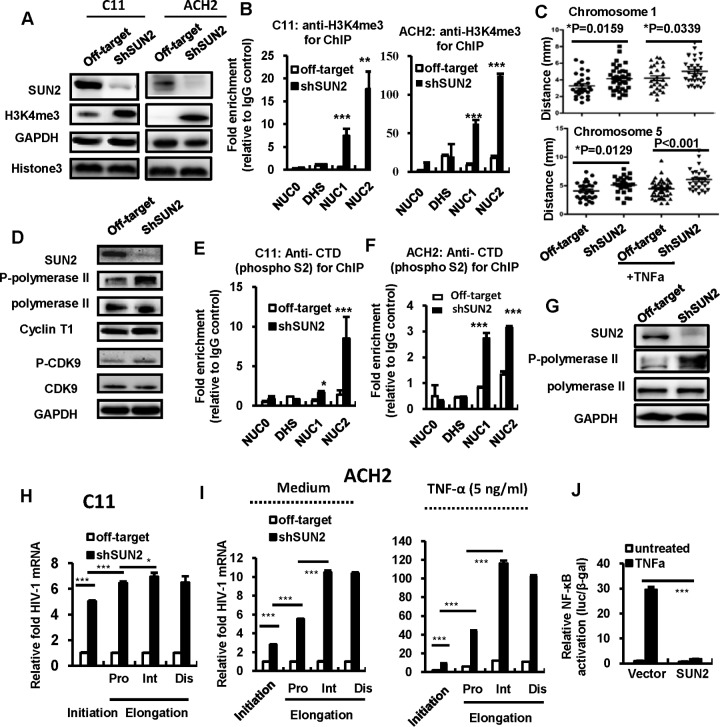
SUN2 maintains the repressive chromatin of the HIV-1 5′-LTR and inhibits both the initiation and elongation of HIV-1 transcription. (A and B) SUN2 knockdown promotes H3K4me3 expression and association with HIV-1 LTR. C11 or ACH2 cells were infected with lentiviruses containing SUN2 shRNA or the off-target control for 72 h, H3K4me3 expression was assessed by Western blotting (A), and the association of H3K4me3 with HIV-1 5′-LTR regions was determined by a cross-linked ChIP assay (B). (C) SUN2 knockdown increases the distance between sister chromatins of chromosomes as detected by FISH assay. C11 cells with or without SUN2 knockdown performed as described above were treated with TNF-α (1 ng/ml) for an additional 24 h. The FISH assay was performed to detect the distances between sister chromatins of chromosomes 1 and 5. Cells were observed under a confocal microscope. (D to G) SUN2 knockdown promotes the Ser-2 phosphorylation (phospho-S2) of RNAPII and its binding with HIV-1 LTR. C11 or ACH2 cells were infected with lentiviruses containing SUN2 shRNA or the off-target control for 72 h, cells were harvested for Western blot immunostaining with the indicated specific antibodies (D and G), and the association of Ser-2-phosphorylated RNAPII at the CTD with HIV-1 5′-LTR regions was determined by a cross-linked ChIP assay (E and F). (H and I) SUN2 knockdown significantly increased HIV 5′-LTR-driven transcription initiation and elongation. C11 or ACH2 cells were infected with lentiviruses containing SUN2 shRNA or the off-target control for 72 h, ACH2 cells were further stimulated with or without TNF-α (5 ng/ml) for an additional 24 h, and total mRNAs were extracted and HIV-1 transcription initiation and the elongated proximal (Pro), intermediate (Int), and distal (Dis) transcripts were assessed by qPCR with specific primers. (J) SUN2 overexpression inhibits NF-κB activation. pCDNA3.1-HA/SUN2 or the vector control and the NF-κB reporter plasmid were cotransfected into HEK293T cells, and the β-galactosidase (β-Gal)-expressing vector pCMV-β-galactosidase was used to normalize transfection efficiency. At 24 h posttransfection, cells were treated with or without TNF-α (5 ng/ml) for an additional 24 h, and then cells were harvested and the reporter gene expressions were assessed. Data are presented as mean ± SD. Results are representative of at least three independent experiments. *, *P* < 0.05, **, *P* < 0.01, and ***, *P* < 0.001, significantly different as determined by an unpaired *t* test.

Multiple lamin-associated chromatin-binding factors can regulate the heterochromatic structure (53–55). The chromatin structure of HIV-1 proviral 5′-LTR is a critical parameter in regulating viral transcription. The formation of heterochromatin structure can result in the repressive chromatin that blocks the transcriptional initiation and elongation (10–13, 56, 57). To investigate whether SUN2-mediated inhibition of LTR activity acted through maintaining repressive chromatin, we performed ChIP analysis of both HIV-1 latently infected C11 and ACH2 cells with a specific antibody against histone H3 (trimethyl K4) (H3K4me3), which is a marker of active chromatin in association with a highly acetylated histone H3 (58, 59). The knockdown of SUN2 not only increased the expression of H3K4me3 but also promoted its recruitment to HIV-1 LTR NUC1 and NUC2 regions in both C11 and ACH2 cells (Fig. 5A and B).

Maintaining distance between homologous chromosomes in human cells could regulate genome stability and gene expression (60). Thus, we also performed fluorescent *in situ* hybridization (FISH) analysis to characterize the chromatin features in C11 cells containing HIV-1 provirus by detecting the distribution of sister chromatin. The SUN2 knockdown significantly increased the distance between sister chromatin of representative chromosomes 1 and 5 (Fig. 5C; see Fig. S3 in the supplemental material).

10.1128/mBio.02408-17.3 FIG S3  SUN2 knockdown increases the distance between sister chromatins of chromosomes as detected by FISH assay. C11 cells were infected with lentiviruses containing SUN2 shRNA or the off-target control for 72 h and then treated with TNF-α (1 ng/ml) for an additional 24 h. The FISH assay was performed to detect the distances between sister chromatins of chromosomes 1 and 5. Cells were observed under a confocal microscope. The arrows indicate the cells in which the changes in chromosome distances are more dramatic. Download FIG S3, TIF file, 0.5 MB. Copyright © 2018 Sun et al. 2018 Sun et al. This content is distributed under the terms of the Creative Commons Attribution 4.0 International license.

The formation of repressive chromatin can block the recruitment of positive transcription factors on the LTR to impede the initiation and elongation of transcription. HIV-1 Tat protein activates viral gene expression through promotion of transcriptional elongation mediated by RNA polymerase II (RNAPII). By binding to the TAR region of HIV-1 5′-LTR, Tat recruits the positive transcription elongation complex (P-TEFb) consisting of cell cycle-dependent kinase 9 (CDK9) and cyclin T1 to enhance phosphorylation of the C-terminal domain (CTD) of RNAPII (61–63). Having demonstrated that SUN2 inhibited HIV-1 Tat-driven transactivation of transcription, we further investigated the effect of SUN2-mediated formation of the repressive chromatin on HIV-1 transcription by characterizing the features of P-TEFb and RNAPII. Though the SUN2 knockdown did not affect the expression of cyclin T1 and CDK9, nor the phosphorylation of CDK9, it increased the Ser-2 phosphorylation (phospho-S2) of RNAPII at the CTD and promoted the association of phosphorylated RNAPII with the HIV 5′-LTR in C11 cells (Fig. 5D and E) and ACH2 cells (Fig. 5F and G). HIV-1 transcription initiation and elongation were assessed by quantitative PCR (qPCR) with specific primers (64). As a consequence of SUN2-mediated formation of repressive chromatin, the SUN2 knockdown significantly increased HIV 5′-LTR-driven transcription initiation and elongation in C11 cells (Fig. 5H) and ACH2 cells (Fig. 5I). Because NF-κB activity is required for initiation of HIV-1 LTR-driven gene transcription, we coexpressed SUN2 and an NF-κB reporter in HEK293T cells and treated the cells with TNF-α to activate NF-κB. The overexpression of SUN2 significantly suppressed TNF-α-induced NF-κB activation (Fig. 5J). Taken together, these data demonstrate that SUN2 maintains the repressive chromatin of HIV-1 5′-LTR and inhibit the initiation and elongation of HIV-1 transcription.

### The reactivation and replication of HIV-1 disassociate SUN2-lamin A/C.

**FIG 6:**
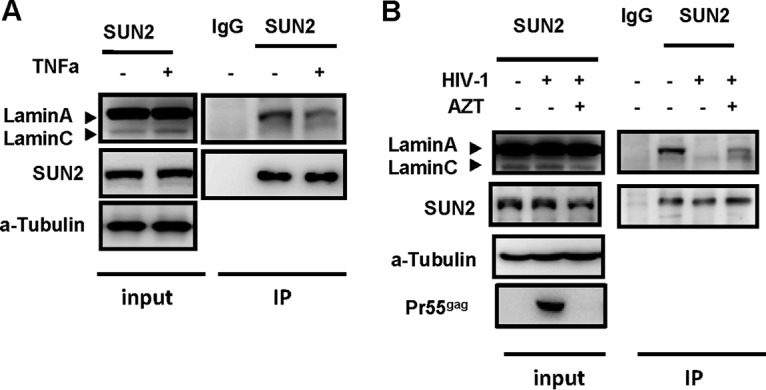
The reactivation and replication of HIV-1 disassociate SUN2-lamin A/C. (A) C11 clones were stimulated with or without 1 ng/ml TNF-α for indicated time. The whole-cell lysates were subjected to immunoprecipitation using anti-SUN2-specific antibody or IgG control, and the immunoprecipitates were separated by SDS-PAGE and analyzed by immunoblotting using these indicated specific antibodies. (B) PHA-P-activated primary CD4^+^ T cells were infected with or without replication-competent HIV-1_NL4-3_ for 5 days, in presence or absence of AZT (20 ng/ml). The whole-cell lysates were immunoprecipitated with anti-SUN2-specific antibody or IgG control, and the immunoblotting was performed as described above.

Having demonstrated the importance of SUN2-lamin A/C association for repressing HIV-1 proviral transcription, we next investigated whether the reactivation and replication of HIV-1 could lead to disassociation of SUN2-lamin A/C. C11 cells were treated with TNF-α to induce HIV-1 reactivation from proviral DNA and used for immunoprecipitation of SUN2 and lamin A/C complex with an anti-SUN2-specific antibody. Results showed that HIV-1 reactivation disassociated SUN2 from lamin A/C (Fig. 6A). Similarly, the infection of replication-competent HIV-1_NL4-3_ in PHA-P-activated primary CD4^+^ T cells disrupted the association between SUN2 and lamin A/C, and the disassociation could be restored when HIV-1 replication was blocked with zidovudine (AZT) (Fig. 6B). Taken together, these data proved that the reactivation and replication of HIV-1 disassociate the SUN2-lamin A/C complex, demonstrating the reversibility of the association of SUN2 with lamin A/C during HIV-1 infection.

## DISCUSSION

**FIG 7:**
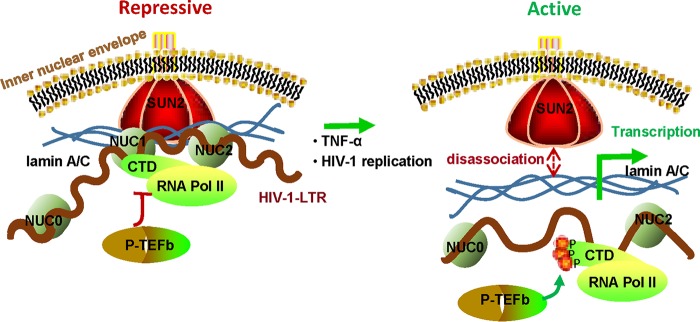
Schematic illustration of the SUN2-lamin A/C interaction that modulates chromatin structure and viral transcription. SUN2 is detained in the perinuclear space and binds to KASH domain-containing nuclear envelope proteins, such as nesprin-1 and nesprin-2. The association with lamin A/C tethers SUN2 to the HIV-1 LTR NUC1 and NUC2 regions to maintain the repressive chromatin, and thus blocks the recruitment of phosphorylated RNAPII and consequently inhibits HIV-1 transcription (left panel). Upon TNF-α stimulation or HIV-1 replication, SUN2 disassociates with lamin A/C, allowing chromatin to become active and permitting viral transcription (right panel). CTD, C-terminal domain of RNA polymerase II (RNAPII); Nuc, nucleosome; P-TEFb, transcription elongation complex.

The reversible silencing of HIV-1 LTR-driven transcription is of paramount importance for an integrated provirus to maintain viral latency (3, 5, 41). A multitude of cellular and viral features that silence HIV-1 transcription and modulate the formation of repressive chromosome represent one of the key areas of intensive investigation (10–13, 56, 57), in which many mechanistic questions remain to be answered. In this study, we revealed a pivotal role of SUN2 as a novel chromatin reassembly factor in modulating chromatin structure and HIV-1 transcription. SUN2 positions to the HIV-1 LTR NUC1 and NUC2 regions via an association with lamin A/C to maintain the repressive chromatin and thus blocks the recruitment of phosphorylated RNAPII, leading to an inhibition of the initiation and elongation of HIV-1 proviral transcription. Upon TNF-α stimulation or HIV-1 replication, SUN2 disassociates with lamin A/C, rendering an active chromatin and permitting viral transcription (Fig. 7).

The overexpression of SUN2 has previously been reported to inhibit HIV-1 infection in some cell lines and primary monocyte-derived dendritic cells (MDDCs) (33, 34), and the mechanism of which was suggested to be a blockage of HIV-1 infection at the early stages between reverse transcription and nuclear entry (33). However, it was uncertain whether endogenous SUN2 also modulates HIV infection. It has been reported that the endogenous SUN2 helps cyclophilin A (CypA) to restrict the nuclear import of HIV strains that have mutations in the CypA binding loop of capsid in murine bone marrow-derived DCs, but not in human MDDCs, THP-1 cells, and primary CD4^+^ T cells (35). However, SUN2 has also been demonstrated to be an essential host factor for promoting the infection of HIV-1 and HIV-2 in human primary CD4^+^ T cells and MDDCs (35). A recent publication demonstrated that 5 days after the silencing of endogenous SUN2 by transduction of shSUN2 in activated primary CD4^+^ T cells, there was impaired cell proliferation, activation, and viability and thereby subsequent HIV infection in these cells (36). In our study, we performed transduction of shSUN2 for 3 days and then infected cells with HIV-1 for an additional 3 days, and found that SUN2 knockdown significantly elevated HIV-1 infection of human primary CD4^+^ T cells, without obviously changing T-cell activation status after the total 6-day incubation, by monitoring the surface expression of CD25 and HLA-DR. The discrepancy may be explained by the fact that SUN2 plays multiple roles in regulating nuclear positioning, centrosome localization, germ cell development, and telomere positioning (65–67), besides its mechanostructural function. The complete knockdown of SUN2 may induce cell apoptosis and consequently decreased viral replication (37).

SUN2 overexpression induces a multilobular nuclear shape and inhibits HIV but does not impact cell viability (33). The nucleoplasmic domain of SUN2 that mediates its interaction with nuclear lamina, but not the SUN domain, is required for the induction of the nuclear change and the inhibition of HIV replication (33). Consistent data have been obtained in our system in that the association of SUN2 with lamin A/C was required for SUN2-mediated inhibition of HIV-1 LTR activity. Moreover, the N-terminus deletion that renders it incapable of associating with lamin A/C, but not the C-terminus deletion, abolished the inhibition of LTR-driven basal gene expression.

Besides providing structural integrity for modulating cytoskeletal organization and nuclear positioning, lamins provide anchoring sites for chromatin domains and regulate chromatin dynamics by associating with numerous host proteins (26–30, 32). Lamin A/C binds both heterochromatin and euchromatin to regulate chromatin organization and transcription (28, 55). SUN2 shows the dependence on lamin A/C for locating to the nuclear envelope (25). The association of SUN2 with lamin A/C may generate a repressive chromatin that blocks the recruitment of positive transcription factors to the HIV-1 5′-LTR. Therefore, upon SUN2 knockdown in HIV-1 latently infected Jurkat T cells, the expression and association of HIV-1 5′-LTR with H3K4me3, a marker of actively transcribed euchromatin, was increased; SUN2 knockdown also increased the Ser-2 phosphorylation of RNAPII at the CTD and promoted the recruitment of phosphorylated RNAPII on the HIV 5′-LTR. Besides SUN2, the other LINC complex-associated nuclear envelope proteins such as emerin have been implicated in regulation of HIV-1 infectivity (68–70).

The stimulation with type I interferon did not appear to induce SUN2 expression (33, 35), although the SUN2 gene was reported as an ISG by a gene screening approach (34). We found that the stimulation of resting CD4^+^ T cells with either PHA-P or the anti-CD3/CD28 antibodies could significantly diminish SUN2 expression, which helps to explain the increased susceptibility of activated primary CD4^+^ T cells for HIV-1 infection.

Intriguingly, we found that the reactivation and replication of HIV-1 were accompanied by the disassociation of the SUN2-lamin A/C complex. The modulation of chromatin dynamic and transcription by the association of SUN2 with lamin A/C may generate a global effect on cells, which could not be completely ruled out in our study. However, we have observed that SUN2 knockdown did not change T-cell activation status markedly, by monitoring the surface expression of CD25 and HLA-DR. Modulation of HIV latency through a global effect on cells is not necessarily a bad thing: several chromatin-regulating host factors that regulate HIV transcription and latency also have such global effects: the chromatin remodeling complex SWI/SNF/BAF has an ATP dependency (7), and the chromatin reassembly factors Spt6 and Chd1 (8, 9) inhibited HIV through some global effect on the cell. In particular, the epigenetic regulation of the HIV proviral 5′-LTR, via the deacetylation modification of histone and the CpG methylation, is predictably acting through their global effects on cells (10, 12, 15, 17, 18, 71). Despite having caused global effects, the epigenetic modifications of histone provide an attractive target for the development of HIV latency reversal agents (LRAs) to purge HIV. For example, the histone deacetylase (HDAC) inhibitor, including three FDA-approved molecules for treatments of T-cell lymphomas, SAHA (vorinostat), belinostat, and romidepsin, represent an important class of HIV LRAs that have been tested in clinical trials (72).

Several HIV-1 latency models, including transformed cell lines and primary cells, have been established, and multiple disparities remain in those latency models regarding the T-cell subtypes being represented, the genetic compositions of the viruses used, and the cellular signaling adopted for reactivation (40). In this study, the transformed Jurkat CD4^+^ T cells, ACH2 cells, and the primary T_CM_ cell model were mainly used to validate this result. Usage of a wider range of other models could be helpful to confirm the role of SUN2 in maintaining HIV-1 latency.

Taken together, our data demonstrate for the first time that SUN2 represses HIV-1 LTR-driven transcription, by associating with lamin A/C to maintain the repressive chromatin. Our finding provides novel insights into SUN2-mediated modulation of HIV-1 infection and facilitates a better understanding of host modulation of HIV-1 latency.

## MATERIALS AND METHODS

### Ethics statement.

The Medical Ethics Review Committee of Institut Pasteur of Shanghai, Chinese Academy of Sciences, approved the usage of human cells.

### Cells.

Human embryonic kidney cells (HEK293T) were cultured in Dulbecco’s modified Eagle’s medium (DMEM) (HyClone) containing 10% fetal bovine serum (FBS) (HyClone) and 100 U/ml penicillin and 100 µg/ml streptomycin. Jurkat and HIV-1 latently infected Jurkat T cells (C11 clone) were provided by Huan-Zhang Zhu (Fudan University, Shanghai, China). ACH2 is a clone of HIV-1 latently infected CD4^+^ CEM cells that contains a single copy of proviral DNA per cell (44, 45) and was provided by Shi-Bo Jiang (Fudan University, Shanghai, China). Jurkat T cells and ACH2 cells were grown in RPMI 1640 medium supplemented with 10% fetal bovine serum (Gibco), 100 U/ml penicillin, and 100 µg/ml of streptomycin (Invitrogen) at 37°C under 5% CO_2_.

Human peripheral blood mononuclear cells (PBMCs) were purchased from Changhai Hospital of Shanghai, China. PBMCs were isolated from the buffy coats of healthy donors by Ficoll density gradient centrifugation as described previously (73). CD4^+^ T cells were further isolated from PBMCs using anti-CD4-specific antibody-coated magnetic beads (Miltenyi Biotec, Inc.) according to manufacturer’s instructions. CD4^+^ T cells were activated with 5 µg/ml phytohemagglutinin-P (PHA-P) (Sigma-Aldrich) or anti-CD3/CD28 antibodies for 48 h and cultured in RPMI 1640 medium with 10% fetal bovine serum (FBS) (HyClone) in the presence of recombinant interleukin-2 (IL-2) (RD Systems). The activation status of cells was monitored by detecting the surface expression of CD25 and HLA-DR. Allophycocyanin (APC)–Cy7–anti-human HLA-DR (LN3; EBioscience)- and APC–anti-CD25 (BC 96, EBioscience)-specific antibodies were used for immunostaining. Cells were detected using a Fortessa flow cytometer (BD Pharmingen) and analyzed with the assistance of FlowJo 7.6.1 software.

### Plasmids and shRNA.

The human full-length SUN2 gene encoding 717 amino acids with a C-terminal hemagglutinin (HA) tag was cloned into the pCDNA3.1 plasmid. The N-terminus deletion [SUN2(335–717)- and SUN2(190–717)-containing mutant] and the C-terminus deletion [SUN2(1–334)- and SUN2(1–189)-containing mutant] were constructed from the pCDNA3.1-HA/SUN2 plasmid by PCR. pGL3-LTR-luc was constructed by PCR amplification of the corresponding DNA fragment from pNL4-3 and subsequently cloned into the pGL3-basic vector (Promega, Madison, WI). pRK-Flag/tat was kindly donated by De-Yin Guo (Wuhan University, China) (74). The 3κB-luc nuclear factor kappa B (NF-κB) reporter plasmid was kindly donated by Chen Wang (Shanghai Institute of Biochemistry and Cell Biology, Chinese Academy of Sciences, Shanghai, China) as described previously (75). Lipofectamine 2000 (Life Technologies, Inc.) was used for transfection according to the manufacturer’s protocol. The targeted sequences of shRNAs have been described previously (76): SUN2 shRNA, 5′ GCC TAT TCA GAC GTT TCA CTT 3′; lamin A/C shRNA, 5′ CAT GGG CAA TTG GCA GAT CAA 3′; and the off-target control, 5′ TTC TCC GAA CGT GTC ACG TAT 3′. SUN2 shRNA was subcloned into the pLKO.1-puro shRNA expression vector. Calcium phosphate-mediated transfection of HEK293T cells was used to generate shRNA lentiviruses as previously described (73). Cell viability was monitored with anti-annexin-V–fluorescein isothiocyanate (FITC) antibody and a propidium iodide (PI) apoptosis detection kit (Invitrogen) and analyzed by flow cytometry.

### Virus stocks.

Calcium phosphate-mediated transfection of HEK293T cells was used to generate virus stock. Pseudotyped single-cycle infectious HIV-Luc/VSV-G or HIV-Luc/NL-3 was cotransfected with the luciferase reporter HIV-1 proviral plasmid pLAI-Δ-env-Luc and the expression plasmid vesicular stomatitis virus G (VSV-G) protein or HIV-1_NL4-3_ Env, and replication-competent HIV-1_NL4-3_ (CXCR4-tropic) virus was transfected with pNL4-3 of HIV-1 proviral vector as previously described (77). Plasmids were kind gifts from Li Wu (The Ohio State University, USA). Harvested supernatants of transfected cells that contained viral particles were filtered and titrated with p24^*gag*^ capture enzyme-linked immunosorbent assay (ELISA). The HIV-1 p24^*gag*^-specific monoclonal antibodies were a kind gift from Yong-Tang Zheng (Kunming Institute of Zoology, Chinese Academy of Sciences, China). Viral infection was measured by detecting luciferase activity using the luciferase assay system (Promega) or Western blotting to detect viral protein expression.

### Real-time RT-PCR.

Total cellular DNA was extracted with a QIAamp DNA minikit (Qiagen, Valencia, CA). Quantitative PCR for *gag* was performed for detection of HIV-1 proviral DNA. Total cellular RNA was extracted using TRIzol reagent (Life Technologies, Inc.) and reverse transcribed into cDNA using the ReverTra Ace qPCR reverse transcription (RT) master mix with gDNA (genomic DNA) Remover (Toyobo). Real-time PCR was performed using the Thunderbird SYBR qPCR mix (Toyobo) on the ABI 7900HT real-time PCR system. The data were analyzed by a SYBR green-based system (Toyobo), semiquantified, and normalized with glyceraldehyde-3-phosphate dehydrogenase (GAPDH). The primers have been described previously (73): *gag*, forward, 5′ GTG TGG AAA ATC TCT AGC AGT GG 3′, and reverse, 5′ CGC TCT CGC ACC CAT CTC 3′; *tat-rev*, forward, 5′ ATG GCA GGA AGA AGC GGA G 3′, and reverse, 5′ ATT CCT TCG GGC CTG TCG 3′; *SUN2*, forward, 5′ AAA CTG CTG CTC GCA TCC 3′, and reverse, 5′ GAG TCT TGC TGA TGC TCT GCT 3′; and GAPDH, forward, 5′ ATC CCA TCA CCA TCT TCC AGG 3′; and reverse, 5′ CCT TCT CCA TGG TGG TGA AGA C 3′.

The integrated HIV-1 proviral DNA was quantified using a two-step Alu PCR, as previously described (78). The following primers and probe were used: primers Alu, forward, 5′ AGC CTC CCG AGT AGC TGG GA 3′, and reverse, 5′ TGC TGG GAT TAC AGG CGT GAG 3′; first *gag*, reverse, 5′ CAA TAT CAT ACG CCG AGA GTG CGC G CTT CAG CAA G 3′; second LTR, forward, 5′ TTG TTA CAC CCT ATG AGC CAG C 3′; and second tag, reverse, 5′ CAA TAT CAT ACG CCG AGA GTG C 3′; and fluorescence-labeled specific probe, 5′-6-carboxyfluorescein (FAM)-ACA CTA CTT GAA GCA CTC AAG GCA AGC TTT-6-carboxytetramethylrhodamine (TAMRA)-3′. The first-round PCR cycle conditions were as follows: a DNA denaturation and polymerase activation step of 10 min at 95°C followed by 12 cycles of amplification (95°C for 15 s, 60°C for 30 s, and 72°C for 5 min). The second-round PCR was performed on the first-round PCR products by using the tag-specific primer (second-tag-R) and the LTR primer (second-LTR-F). PCR was performed using the Goldstar TaqMan mixture (CWBIO, China) with predenaturation at 95°C for 10 min, amplification with 45 cycles of denaturation (95°C for 15 s), and annealing (60°C for 60 s) on the ABI 7900HT real-time PCR system.

### ChIP.

ChIP experiments were performed according to protocol provided by EZ-ChIP chromatin immunoprecipitation kit (Millipore) (43, 73). Briefly, C11 cells were cross-linked with 1% formaldehyde for 10 min at room temperature and quenched with 0.125 M glycine for 5 min. After lysis, nuclear extracts were separated and chromatin was sheared by sonicator (Bioruptor UCD-200; Diagenode) for 10 min (10 s on and 10 s off) on ice to obtain DNA fragments of 200 to 1,000 bp in length. One percent of total sheared chromatin DNA was used as the input. Nuclear extracts were incubated with the indicated antibodies at 4°C overnight. Protein G/A-labeled Dynabeads were added to each sample at 4°C for 2 h for immunoprecipitation. The immunoprecipitated DNA was analyzed by a real-time PCR (ABI Prism 7900 real-time PCR system) at 40 cycles with Thunderbird SYBR qPCR mix (Toyobo). The primers targeting the HIV LTR NUC0, DHS, NUC1, and NUC2 regions have been described previously (7): NUC0, forward, 5′ TGG ATC TAC CAC ACA CAA GG 3′, and reverse, 5′ GTA CTA ACT TGA AGC ACC ATC C 3′; DHS, forward, 5′ AAG TTT GAC AGC CTC CTA GC 3′, and reverse, 5′ CAC ACC TCC CTG GAA AGT C 3′; NUC1, forward, 5′ TCT CTG GCT AAC TAG GGA ACC 3′, and reverse, 5′ CTA AAA GGG TCT GAG GGA TCT C 3′; and NUC2, forward, 5′ AGA GAT GGG TGC GAG AGC 3′, and reverse, 5′ ATT AAC TGC GAA TCG TTC TAG C 3′.

### Immunoprecipitation and immunoblotting.

Cells were lysed in radioimmunoprecipitation assay (RIPA) buffer (50 mM HEPES, pH 7.4, 150 mM NaCl, 0.5 mM EGTA, 1% protease inhibitor cocktail [Sigma], 1 mM sodium orthovanadate, 1 mM NaF, 1% [vol/vol] Triton X-100, 10% [vol/vol] glycerol) for 1 h on ice with brief vortexing every 10 min. After centrifugation for 10 min at 12,000 × *g*, the lysates were incubated with the indicated antibody at 4°C overnight. Protein G/A-labeled Dynabeads were added into each sample at 4°C for 2 h for immunoprecipitation. The immunoprecipitates were separated by SDS-PAGE and analyzed by immunoblotting. For immunoblotting, cells were lysed for 1 h at 4°C in ice-cold RIPA buffer. After centrifugation for 10 min at 12,000 × *g*, supernatant was boiled in reducing SDS sample loading buffer and analyzed by SDS-PAGE. Specific primary antibodies were used, followed by horseradish peroxidase-conjugated goat anti-mouse IgG or goat anti-rabbit IgG (Sigma) as the secondary antibodies. Five percent of total lysates was used as the input.

The following antibodies were used for ChIP or immunoblotting: anti-SUN2 (ab133591; Abcam, Inc.), anti-lamin A/C (sc-7292x; Santa Cruz), anti-HA tag (26D11; Abmart, Inc., Shanghai, China), anti-cyclin T1 (81464; Cell Signaling Technology, Inc.), anti-cyclin-dependent protein kinase 9 (anti-CDK9) (2316; Cell Signaling Technology, Inc.), anti-phospho-CDK9 (Thr186) (2549; Cell Signaling Technology, Inc.), anti-RNA polymerase II (05-952; Merck Millipore), anti-RNA polymerase II C-terminal domain (CTD) repeat YSPTSPS (phospho-S2) (ab5095; Abcam, Inc.), anti-H3K4me3 (histone H3 trimethyl K4) (ab8580; Abcam, Inc.), anti-α-tubulin (T6199; Sigma-Aldrich), and anti-GAPDH (M20006; Abmart Inc., Shanghai, China).

### Confocal microscopy.

HEK293T cells were seeded on poly-l-lysine-coated microscope slides and transfected with SUN2 plasmid or the mutants. Cells were fixed and immunostained with an anti-HA or anti-lamin A/C antibody, followed by secondary Alexa 555-labeled anti-mouse or Alexa 488-labeled anti-rabbit IgG antibodies. The nucleus was labeled with DAPI (4′,6-diamidino-2-phenylindole). Slides were mounted with Dako fluorescent mounting medium and observed using a laser scanning confocal microscope (Leica SP5).

### FISH.

FISH was performed according to protocol of the FISH assay kit (Abnova, Taiwan). Briefly, cells were placed on slides, and sequentially treated with methanol, acetic acid, 2× standard saline citrate, hot citric acid, and pepsin. Centromere-specific α-satellite (CEN) probes for chromosomes 1 (Texas Red), and 5 (Cy5) were added to each slide, the DNA and probe were codenatured and hybridized, and the slides were washed and counterstained. Cells were then viewed with a Leica SP8 fluorescence microscope. For each chromosome, >30 nuclei were analyzed.

### Assays for HIV-1 reactivation from latently infected cells.

HIV-1 latently infected Jurkat T-cell C11 clone or ACH2 cells were transduced with either shSUN2 or the off-target control for 72 h and then stimulated with TNF-α (1 ng/ml), PMA (Sigma) (1 nM), or PMA-ionomycin for an additional 24 h. The GFP-positive populations of C11 cells were detected using a Fortessa flow cytometer (BD Pharmingen) and analyzed with the FlowJo 7.6.1 software. Viral reactivation in ACH2 was detected by quantifying the produced viral particles in supernatant with p24^*gag*^ capture ELISA or the cell-associated *gag* mRNA.

We also used the primary T_CM_ cell model established by Vicente Planelles’s lab (51, 52) with slight modifications. Briefly, naive CD4^+^ T cells were isolated by MACS microbead-negative sorting using the naive T-cell isolation kit (Miltenyi Biotec, Inc.). Naive CD4^+^ T cells were stimulated with anti-CD3/CD28 antibody-coated microbeads for 3 days. The microbeads were then removed, and cells were further cultured for an additional 4 days. Cells were infected with VSV-G-pseudotyped defective HIV (DHIV)-GFP (dEnv) and cultured for an additional 7 days to establish latency. Latently infected cells were infected with lentiviruses containing SUN2 shRNA or the off-target control for 3 days and then stimulated with or without anti-CD3/CD28 antibody-coated microbeads for an additional 3 days for detection of reactivation from latency by either flow cytometry or immunoblotting.

### Statistical analysis.

Statistical analysis was performed using a paired or unpaired Student’s *t* test with SigmaStat 2.0 (Systat Software, Inc., San Jose, CA, USA) or Wilcoxon’s signed-rank test when indicated.
