# Supplementary figures and images for: SUN2 Modulates HIV-1 Infection and Latency through Association with Lamin A/C To Maintain the Repressive Chromatin
Source: mBio. 2018 May 1;9(3):e02408-17. doi: 10.1128/mBio.02408-17 (PMC5930302; doi:10.1128/mBio.02408-17)

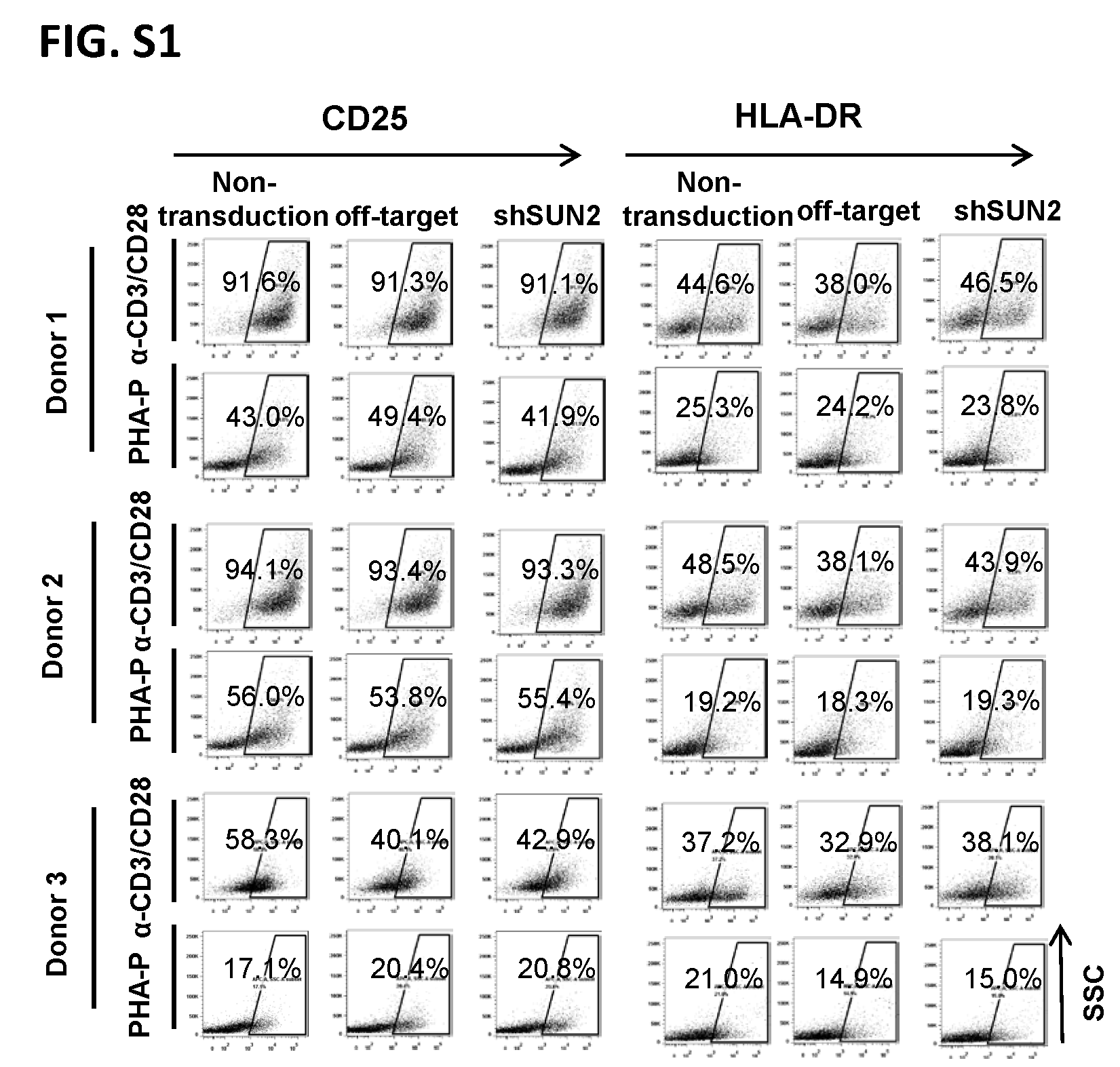

Supplement: FIG S1 [file mbo002183861sf1.tif]

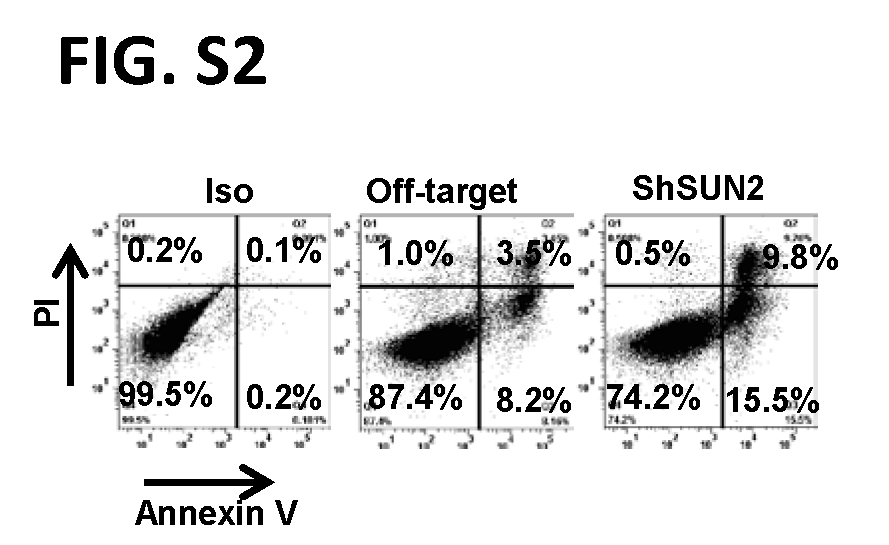

Supplement: FIG S2 [file mbo002183861sf2.tif]

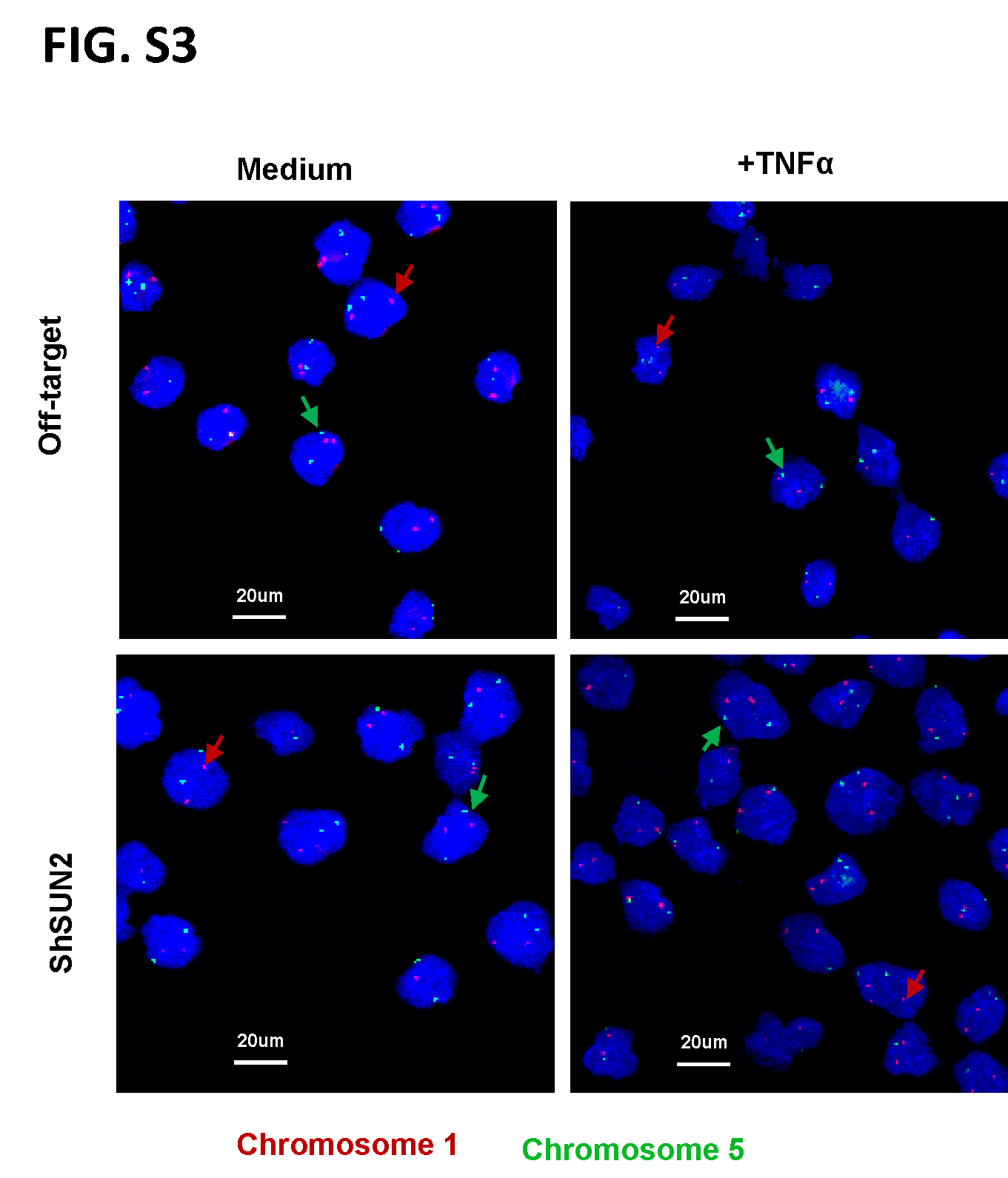

Supplement: FIG S3 [file mbo002183861sf3.tif]
